# Supplementary material for: Anti-inflammatory and Anti-oxidant Activity of Hidrox® in Rotenone-Induced Parkinson’s Disease in Mice
Source: Antioxidants (Basel). 2020 Sep 3;9(9):824. doi: 10.3390/antiox9090824 (PMC7576486; doi:10.3390/antiox9090824)
Supplement: Supplementary file 1 [file antioxidants-09-00824-s001.docx]

Anti-Inflammatory and Anti-Oxidant Activity of Hidrox^®^ in Rotenone-Induced Parkinson’s Disease in Mice

Rosalba Siracusa ^1,†^, Maria Scuto ^2,†^, Roberta Fusco ^1^, Angela Trovato ^2,^*, Maria Laura Ontario ^2^, Roberto Crea ^3^, Rosanna Di Paola ^1,^*, Salvatore Cuzzocrea ^1,4,‡^ and Vittorio Calabrese ^2,‡^

^1^ Department of Chemical, Biological, Pharmaceutical and Environmental Sciences, University of Messina, Viale Ferdinando Stagno D’Alcontres, 31, 98166 Messina, Italy; rfusco@unime.it

^2^ Department of Biomedical and Biotechnological Sciences, University of Catania, Via S. Sofia, 89, 95123 Catania, Italy; ti.lrsoiratno@oiratno.arualairam

^3^ Oliphenol LLC., 26225 Eden Landing Road, Unit C, Hayward, CA, 94545, USA; ppontoniere@creagri.com

^4^ Department of Pharmacological and Physiological Science, Saint Louis University School of Medicine, Saint Louis, 63104, USA

***** Correspondence: trovato@unict.it (A.T.); dipaolar@unime.it (R.D.P.); Tel.: +39-09-5478-1165 (A.T.); +39-09-0676-5208 (R.D.P.)

† Rosalba Siracusa and Maria Scuto are the co-first authors.

‡ Salvatore Cuzzocrea and Vittorio Calabrese shared senior authorship.


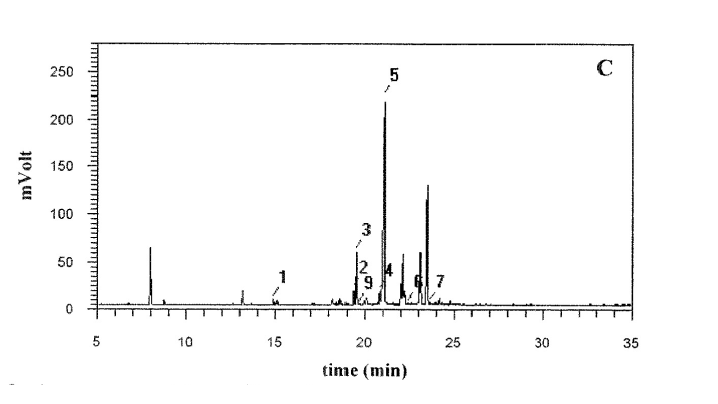


Figure S1. Gas chromatography mass spectrometry (GC-MS) analysis and phenolic compound identification, by spectrum matching and library searching in the NIST/EPS/NIH Mass Spectral Library Database, of HD. Peaks: 1, tyrosol; 2, vanilic acid; 3, hydroxytyrosol; 4, 3,4-dihydroxybenzoic acid; 5, citric acid (hydrolysis acid to obtain HD from OVW, olive aqueous vegetation water); 6, syringic acid; 7, gallic acid; 8, caffeic acid (trace); 9, 3-hydroxy,4-methoxyphenylacetic acid; 10, gentisic acid (trace)


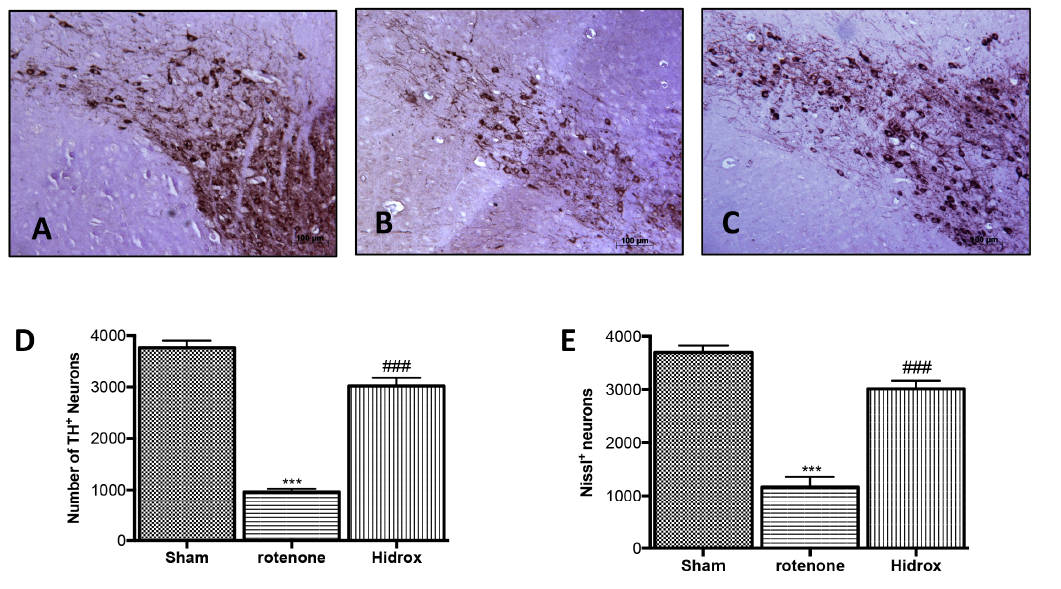
 Figure S2. Stereological analysis in SN of rotenone-treated mice. Sham (A), rotenone-mice (B), and HD-treated mice (C), respectively, stained for stereological counting of TH-positive and cresyl violet positive neurons in sections of the SN from one hemisphere (D,E). Each data is expressed as a number of TH^+^ and Nissl^+^ neurons and are mean ± SEM from 5 mice/group. *** *p* < 0.001 vs. Sham; *** *p* < 0.001 vs. rotenone (D,E). Scale bar 100 μm.


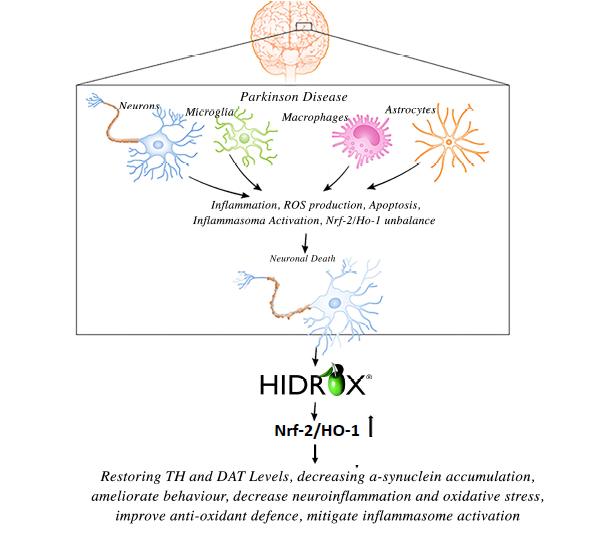
 Figure S3. Processes implicated in the pathogenesis of Parkinson’s and effects of Hidrox^®^.
